# Using routinely recorded data in the UK to assess outcomes in a randomised controlled trial: The Trials of Access

**DOI:** 10.1186/s13063-017-2135-9

**Published:** 2017-08-23

**Authors:** G. A. Powell, L. J. Bonnett, C. Tudur-Smith, D. A. Hughes, P. R. Williamson, A. G. Marson

**Affiliations:** 1 Department of Molecular and Clinical Pharmacology, Clinical Sciences Centre, Lower Lane, Fazakerley, Liverpool, L9 7LJ UK; 2 Department of Biostatistics, University of Liverpool, Waterhouse Building, Block F, 1-5 Brownlow Street, Liverpool, L69 3GL UK; 3 Centre for Health Economics and Medicines Evaluation, Institute of Medical and Social Care Research, College of Health and Behavioural Sciences, Bangor University, Ardudwy, Normal Site, Gwynedd, North Wales LL57 2PZ UK

**Keywords:** Routine data, Administrative data, Feasibility, Data collection

## Abstract

**Background:**

In the UK, routinely recorded data may benefit prospective studies including randomised controlled trials (RCTs). In an on-going study, we aim to assess the feasibility of access and agreement of routinely recorded clinical and non-clinical data compared to data collected during a RCT using standard prospective methods. This paper will summarise available UK routinely recorded data sources and discuss our experience with the feasibility of accessing routinely recorded data for participants of a RCT before finally proposing recommendations for improving the access and implementation of routinely recorded data in RCTs.

**Methods:**

Setting: the case study RCT is the Standard and New Antiepileptic Drugs II (SANAD II) trial, a pragmatic, UK, multicentre, phase IV RCT assessing the clinical and cost-effectiveness of antiepileptic drug treatments for newly diagnosed epilepsy.

Participants: 98 participants have provided written consent to permit the request of routinely recorded data.

Study procedures: routinely recorded clinical and non-clinical data were identified and data requested through formal applications from available data holders for the duration that participants have been recruited into SANAD II. The feasibility of accessing routinely recorded data during a RCT is assessed and recommendations for improving access proposed.

**Results:**

Secondary-care clinical and socioeconomic data is recorded on a national basis and can be accessed, although there are limitations in the application process. Primary-care data are recorded by a number of organisations on a de-identified basis but access for specific individuals has not been feasible. Access to data recorded by non-clinical sources, including The Department for Work and Pensions and The Driving and Vehicle Licensing Agency, was not successful.

**Conclusions:**

Recommendations discussed include further research to assess the attributes of routinely recorded data, an assessment of public perceptions and the development of strategies to collaboratively improve access to routinely recorded data for research.

**Trial registration:**

International Standard Randomised Controlled Trials, ISRCTN30294119. Registered on 3 July 2012. EudraCT No: 2012-001884-64. Registered on 9 May 2012.

## Background

There is a plethora of individual-level, routinely recorded data in the UK. These data are recorded to fulfil specific, defined purposes and are regulated for security, confidentiality and disclosure by The Data Protection Act 1998 [1] and The Freedom of Information Act 2000 [2]. Access to routinely recorded data for ‘secondary purposes’, such as clinical research, is permitted providing that there is demonstrable secondary benefit.

The potential for routinely recorded data to inform clinical research and Health Technology Assessment (HTA) has long been recognised [3]. Presently, there are a number of sources of routinely recorded primary and secondary-care clinical data with regional or national coverage. However, limitations with accuracy of coding, confidentiality, ownership and data access have been previously identified as significant barriers to using routinely recorded data in research [4].

There are numerous examples of retrospective, observational, record-linkage population studies where routine sources have proved a valid and efficient method for providing data for clinical research [5]. In the context of prospective research, such as randomised controlled trials (RCTs), routinely recorded data have been used to inform judgements about the feasibility of sample size and recruitment targets [6] and measuring participant outcomes [3, 7]. Pragmatic cluster RCTs have been coordinated through routine data sources including patient recruitment, randomisation, and administration of intervention and trial assessments, such as through the Clinical Practice Research Datalink (CPRD) [8]. The majority of RCTs incur costs as clinicians assess participants, record outcomes and complete Case Report Forms – hence, using routinely recorded data may provide an efficient alternative method for data collection in addition to reducing the burden on participants. Furthermore, data from non-clinical routine sources may inform outcomes beyond the standard RCT assessments of clinical efficacy and effectiveness. For example, cost data (such as use of health care resources) and socioeconomic data (such as employment and means-tested benefits data) may inform health economic analyses and the assessment of the broader societal impact of health care interventions.

The potential benefits of using routinely recorded data in clinical research have resulted in a political drive to increase implementation, detailed in *The Plan for Growth* [9] and *The NHS Constitution* [10], where research is presented as a core activity making the link explicit between the provision of NHS services and research. Consequently, initiatives, such as the Administrative Data Research Network [11], have been established to provide a method of access to individual-level data, linking clinical and non-clinical sources of routinely recorded data.

The objective of this paper is to review relevant sources of routinely recorded data for England, Scotland and Wales and to discuss our experience with the feasibility of accessing individual-level data for a subgroup of participants enrolled into a RCT before finally proposing recommendations for improving the access and implementation of routinely collected data in RCTs. This is an on-going study and in a future publication we aim to assess the agreement of routinely recorded data compared to paired data collected in a RCT using standard prospective methods.

## Methods

The case study RCT is the Standard and New Antiepileptic Drugs (SANAD) II trial. SANAD II is a pragmatic, UK, multicentre, phase IV RCT funded by the National Institute for Health Research (NIHR) Health Technology Assessment (HTA) programme, assessing the clinical and cost-effectiveness of a number of antiepileptic drugs as first-line treatments for newly diagnosed epilepsy. Data for clinical outcomes, including seizure freedom and adverse events, are recorded on Case Report Forms by the treating clinical team during outpatient appointments. Data to inform cost-effectiveness analyses, including health care resource use and quality of life, are recorded through participant completion of questionnaires. SANAD II is currently recruiting and is expected to report in 2019.

Following research ethics and governance approvals, 470 participants enrolled in SANAD II were invited to provide written consent to permit the request of routinely recorded data for the duration of their participation in SANAD II. Ninety-eight (20.9%) participants provided consent and were included in the study. Relevant sources of routinely recorded data were identified and detailed scoping discussions ensued. Subsequently, where accessible, routinely recorded data for participants recruited into SANAD II were requested through formal applications. The routinely recorded data sources included in this study are as follows:

- Clinical routine data sources: secondary care:
  - ◦ The Health and Social Care Information Centre (HSCIC)
  - ◦ The NHS Wales Informatics Service (NWIS)
  - ◦ The NHS National Services Scotland; Information Services Division (ISD)
- Clinical routine data sources: primary care:
  - ◦ The Clinical Practice Research Datalink (CPRD)
  - ◦ ResearchOne
  - ◦ QResearch
  - ◦ The Health Improvement Network (THIN) database
  - ◦ North West eHealth (NWEH)
- Non-clinical routine data sources:
  - ◦ The Office for National Statistics (ONS)
  - ◦ HM Revenue and Customs (HMRC)
  - ◦ The Department for Work and Pensions (DWP)
  - ◦ The Driver and Vehicle Licensing Authority (DVLA)
- ‘Linked’ routine data sources:
  - ◦ The Secure Anonymised Information Linkage (SAIL) databank
  - ◦ The Administrative Data Research Network (ADRN)

In a future publication, the agreement between routinely recorded data and data collected using standard prospective methods will be assessed for baseline variables such as gender, age and date of first seizure, and for outcome measures relevant to SANAD II such as time to 12-month remission from seizures. To assess agreement between paired continuous data, Bland-Altman methods will be employed. Acceptable clinical limits of agreement for each variable or SANAD II outcome will be specified a priori and compared to the 95% confidence limits of agreement. To assess agreement between paired, nominal categorical datasets, cross tabulations will be constructed followed by calculation of Cohen’s Kappa.

## Results

### Clinical routine data sources: secondary care

Electronic medical records of patients’ use of secondary-care services in the UK are routinely managed on a national basis. A number of public service organisations provide national information, data and IT systems for commissioners, analysts and clinicians in health and social care. Data are recorded to inform patient care, provide the data for remuneration for hospital trusts and are subsequently used to monitor and improve clinical services through clinical research. Table 1 summarises the data sources where access to individual-level data is possible.

**Table 1:** Example sources of routinely recorded secondary-care data

*The Health and Social Care Information Centre* (*HSCIC*) [21] *Data access for clinical research*: *The Data Access Request Service* provides a method of access to a number of routinely collected datasets for England. *Hospital Episode Statistics* (HES) provides clinical, health and socioeconomic data for all secondary-care attendances in England. Datasets include *Accident and Emergency, Admitted Patient, Outpatient, Adult Critical Care, Maternity* and selected *Patient Reported Outcome Measures*. *Previous experience in clinical research*: HES data have been accessed for retrospective linkage studies [22] and to provide data for prospective studies; for example, estimation of health care resource use or measuring outcomes such as long-term mortality [23]
*The NHS Wales Informatics Service* (*NWIS*) [24] *Data access for clinical research*: Data access can be facilitated through *The Public Health Wales Observatory*. The *Patient Episode Database for Wales* (*PEDW*) provides clinical, health and socioeconomic data for all secondary-care attendances in Wales and is broadly comparable to the Admitted Patient HES dataset, with data regarding elective and emergency admissions and maternity care recorded. Additional datasets of relevance to this study include the *Emergency Department* and *Outpatient Datasets.* *Previous experience in clinical research*: PEDW data have been accessed for retrospective analyses; for example, analysis of the incidence of obstetric complication rates [25]
*The NHS National Services Scotland; Information Services Division* (*ISD*) [26] *Data access for clinical research*: The *electronic Data Research and Innovation Service* (*eDRIS*) provides a method of access to ISD datasets including *Outpatient, General Acute/Inpatient, Emergency Department, Unscheduled Care, GP Out of Hours* and *The Prescribing Information System*. Clinical, health and socioeconomic data are recorded and datasets are largely comparable to HSCIC HES. *Previous experience in clinical research*: ISD data have been accessed for retrospective linkage studies; for example, analysis of the incidence of gastrointestinal bleeding and complications including mortality [27]

### Clinical routine data sources: primary care

Electronic medical records of patients’ use of primary-care services in the UK are recorded routinely by the general practitioner to inform patient care and remuneration, but are not currently available for clinical research on a national basis. A number of organisations represent collaborations between governmental bodies or academic institutions and providers of primary-care IT systems. Access on a regional basis is possible through a number of data sources summarised in Table 2.

**Table 2:** Example sources of routinely recorded primary-care data

*The Clinical Practice Research Datalink* (*CPRD*) [28] *Data access for clinical research*: CPRD is a governmental research service jointly funded by the NHS National Institute for Health Research and the Medicines and Healthcare products Regulatory Agency. Following approval by the *Independent Scientific Advisory Committee*, CPRD provides access to de-identified primary-care clinical, health and socioeconomic data for a geographically representative 13 million patients in England for health care research. *Previous experience in clinical research*: CPRD data have been used in retrospective studies for estimating health care resource use, prescription medicines and clinical outcomes [22]. Gulliford conducted two cluster-randomised trials using CPRD: one aimed to reduce inappropriate antibiotic prescribing for acute respiratory infection; the other aimed to increase physician adherence with secondary prevention interventions after first stroke [8]
*ResearchOne* [29] *Data access for clinical research*: ResearchOne is a collaboration between The University of Leeds and The Phoenix Partnership (TTP), developers of the SystmOne clinical database and IT system. De-identified clinical, health and socioeconomic data are available from primary, secondary and out-of-hours care settings for approximately 26 million patients in the UK. *Previous experience in clinical research*: ResearchOne data have been used in public health surveillance studies, retrospective studies [29] and, currently, in combination with CPRD data to measure the outcomes of a cluster RCT [30]
*QResearch* [31] *Data access for clinical research*: QResearch is a collaboration between The University of Nottingham and the developers of the EMIS IT systems*.* De-identified clinical, health and socioeconomic data are available for approximately 18 million patents in the UK. *Previous experience in clinical research*: QResearch data have been used to measure clinical outcomes in case-control and cohort studies [32]
*The Health Improvement Network* (*THIN*) *Database* [33] *Data access for clinical research*: THIN is a collaboration between IMS Health and In Practice Systems, developers of the IT software Vision. De-identified clinical, health and socioeconomic data are available for approximately 11.1 million patients in the UK. *Previous experience in clinical research*: THIN data have been accessed to measure clinical outcomes in cohort and case-control studies [34]
*North West eHealth* (*NWEH*) [35] *Data access for clinical research*: NWEH is a collaboration between The University of Manchester, Salford Royal Foundation Trust and Salford Clinical Commissioning Group*.* NWEH has developed the methodology and governance framework to implement the *Salford Integrated Record*, an integrated primary- and secondary-care electronic medical record, into research as part of the Salford Lung Study [14]. The infrastructure permits access to secondary-care electronic medical records accessed through the HSCIC *Secondary Uses Service*. With participant and GP practice enrolment and consent, the Apollo [36] and Graphnet [37] data-extraction tools are employed to extract participant primary-care electronic medical records that can then be linked to data regarding secondary care. North West eHealth is unique in that data are not de-identified and, therefore, participant consent is required. Furthermore, GP practice enrolment and consent is required to permit the installation of third-party software on their systems and subsequent extraction of data. *Previous experience in clinical research*: NWEH offers a number of primary-care research tools including a randomised controlled trial (RCT) recruitment feasibility assessment, but does not currently routinely provide a bespoke primary-care data-extraction service for research. However, the methodology for this process has been demonstrated [14]

### Non-clinical routine data sources

Non-clinical, individual-level data are routinely recorded by a number of UK governmental departments for a variety of indications. Selected organisations record data that would be informative to prospective clinical research in epilepsy and other diseases, summarised in Table 3.

**Table 3:** Example sources of routinely recorded non-clinical data

*The Office for National Statistics* (*ONS*) [38] *Data access for clinical research*: The ONS records individual-level mortality data and aggregate economic and societal statistics that may inform clinical and health economic analyses. Mortality data can be requested through application to the HSCIC DARS. Aggregate data can be accessed via services provided by ONS such as NOMIS [39] and Data for Neighbourhoods and Regeneration [40]*.* The smallest reported level is the Lower Layer Super Output Area (LSOA) consisting of a population of 1000–3000. *Previous experience in clinical research*: ONS mortality data have been accessed to measure mortality in retrospective and prospective studies [23]
*HM Revenue and Customs* (*HMRC*) [41] *Data access for clinical research*: HMRC is the UK’s national tax authority and responsible for taxation including National Insurance and student loan repayments and the administration of tax credits, child benefit and statutory sick and maternity pay. Individual-level data on employment and tax contributions are recorded and likely to inform health and socioeconomic analyses. The *HMRC Datalab* provides a means to access de-identified, aggregate HMRC data for research. An application, once ‘approved researcher’ status has been gained, must benefit the listed functions of the HMRC. *Previous experience in clinical research*: There was no evidence of individual-level, HMRC data being accessed for clinical research in a scoping search performed in MEDLINE via OVID
*The Department for Work and Pensions* (*DWP)* [42] *Data access for clinical research*: The DWP is responsible for welfare including the provision of state pensions, benefits and child maintenance*.* Individual-level data regarding employment and welfare are likely to inform health and socioeconomic analyses and de-identified, aggregate data are available for social research. *Previous Experience in Clinical Research*: There was no evidence of individual-level, DWP data being accessed for clinical research in a scoping search performed in MEDLINE via OVID
*The Driver and Vehicle Licensing Authority* (*DVLA*) [43] *Data access for clinical research*: The DVLA is responsible for the licensing of drivers and vehicles in the UK and issuing, reviewing and maintaining guidance regarding driving licence status in the context of medical diagnoses*.* The legal requirement for driving licence holders to inform the DVLA of the occurrence of seizures and, subsequently, to regain normal driving privileges after a specified period of seizure freedom raises the possibility of DVLA providing an accurate data source to inform the clinical outcome measures in epilepsy research. *Previous experience in clinical research*: The DVLA publish limited de-identified, aggregate datasets for research, usually involving driving restrictions. There was no evidence of individual-level, DVLA data being accessed for clinical research in a scoping search performed in MEDLINE via OVID

### ‘Linked’ routine data sources

In order to provide a ‘complete’ dataset of the information required to meet research objectives, data from a number of organisations may need to be accessed. This is typically accomplished by linking data sources using identifiers such as patients’ name, date of birth, National Insurance number or NHS number. In response to the growing recognition of the potential of routinely recorded data, initiatives have been established to assist with the provision of linked, de-identified, aggregate data between data sources:

- *The Secure Anonymised Information Linkage* (*SAIL*) *Databank* is an initiative developed by Swansea University and funded by the Welsh Government. SAIL provides a method of access to individual-level, routinely recorded, de-identified electronic data for patients across Wales to support research [12]. Access to clinical datasets provided by NWIS is complemented with numerous non-clinical administrative datasets including births, deaths and demographic data. Following the scoping process a formal application is submitted to the *Information Governance Review Panel* before access to data is granted. SAIL data have been accessed to measure clinical outcomes in retrospective research [13]
- *The Administrative Data Research Network* (*ADRN*) is a UK-wide partnership between universities, government departments, national statistics authorities, funders and researchers, funded by the *Economic and Social Research Council*. ADRN provides a method of access to a number of non-clinical administrative routine datasets including employment, socioeconomic, crime and education data [11] in addition to clinical datasets detailed previously such as those recorded by HSCIC. Following development of a project proposal a formal application is reviewed by the *Approvals Panel* before access to data is granted

### Challenges and feasibility of access

We have requested access to routinely recorded data for individuals enrolled in the SANAD II RCT, resident in England and Wales, who have provided written consent. There were insufficient participants meeting the eligibility criteria resident in Scotland. Data sources were identified and scoping discussions informed the initial assessment of feasibility. Data sources were deemed feasible if individual-level data could be provided for specified individuals providing consent. Resources required including cost and researcher time were also factors important in the assessment of feasibility. Including the preparation, research ethics and governance approval and submission of the applications for data access, significant researcher time and a period of 18 months were required. The feasibility, timeline and key milestones involved for each data source are summarised in Table 4.

**Table 4:** Summary of key application milestones

Routine data source	Summary of key application milestones	Cost structure
The Health and Social Care Information Centre (HSCIC)	*August 2015*: first request to review Participant Information Sheet (PIS) and Consent Form. Sent by enquiries desk to the Data Access Request Service (DARS) *4 November 2015*: second request to review PIS and Consent Form. Sent by enquiries desk to Data Access and Information Sharing Team (DAIS) *23 November 2015*: no feedback yet received. PIS and Consent Form discussed with a member of the DARS team in person at a HSCIC engagement event. Informed that a full, formal application would be required in order for HSCIC to provide feedback on the PIS and Consent Form. This was completed and submitted on 26 November *7 December 2015*: response regarding PIS and Consent Form. Informative teleconference with a member of the DARS team *22 December 2015*: response from the DAIS team in response to the second request on 4 November 2015. Teleconference provided feedback, in agreement with that received from the DARS team on 7 December *29 February 2016*: as directed by HSCIC, submission of a new formal application using the existing application process *18 April 2016*: formal acknowledgment of submission. Requested to submit the application via the DARS Online Portal *22 April 2016*: formal application submitted via DARS Online Portal *24 May 2016*: Data Access Advisory Group (DAAG) review. Caveats to be addressed before approval *26 May 2016*: caveats addressed, application updated and re-submitted *13 July 2016*: DAAG approved. Hospital Episode Statistics (HES) data available for download	Standard cost recovery structure applied: *£1000 new application* *£900 release fee* *£500 3-year agreement* *£300 per dataset per year*
The Secure Anonymised Information Linkage Databank (SAIL)	*22 April 2015*: first contact regarding application process and association with the Administrative Data Research Network (ADRN) *June 2015*: informative teleconference regarding the SAIL application process and scoping procedure *7 July 2015*: protocol regarding methods specific to SAIL submitted *August 2015*: request to review PIS and Consent Form. Sent to information governance officer for review *September 2015*: feedback on PIS and Consent Form from information governance officer. Scoping document issued by SAIL *January 2016*: final review of PIS/Consent Form requested following revisions required for the other data sources *February 2016*: submission of full, formal application *March 2016*: feedback received following internal review with amendments suggested *April 2016*: application re-submitted for formal Information Governance Review Panel (IGRP) review, outcome pending	Standard cost recovery structure applied: *£500 base cost* *£291 data transfer to SAIL* *£1455 individual-level data processing* *£500 data transfer*
The Clinical Practice Research Network (CPRD)	*November 2014*: first contact regarding feasibility of the study, response received broadly confirming feasibility *August 2015*: following protocol development, further contact regarding feasibility. Informed by CPRD that the Confidentiality Advisory Group and ethical approvals with HSCIC need to be updated to permit identifiable, linked data release and the timelines to resolve these are unclear. Furthermore, informed that compliance with HSCIC’s governance framework needs to be approved. No further contact as the issues with linked data release, cost and population coverage make CPRD not feasible for inclusion in this study	Standard cost recovery structure applied: *£7500 CPRD GOLD for <1000 patients* *£4250 linked HES inpatient* *£850 linked HES outpatient* *£3000–5000 extraction, specification, assurance*
QResearch | ResearchOne The Health Improvement Network (THIN) Database	*September 2015*: all organisations contacted. Confirmed that data are de-identified only, with no facility to re-identify patients as would be needed for this study. Data sources are, therefore, not feasible for inclusion in this study	N/A
North West eHealth	*October 2015*: first contact, the service is not routinely offered but feasibility of the process broadly confirmed *November 2015*: correspondence via email to request review of the protocol, PIS and Consent Form, confirm the methodology and determine provisional costings. Further discussion during a face-to-face meeting at NWEH *December 2016*: discussion with the third party, Apollo Medical Software Solutions, regarding the development of the data query to permit the extraction of data. Response received confirming the structure of the existing data query can be used for GP practices in Salford already holding a data-sharing agreement with NWEH, but a bespoke query would be required for this study *January 2016*: final review of PIS/Consent Form requested and received *May 2016*: < participants consented to inclusion in the study are registered in eligible GP practices; therefore, accessing data through NWEH is not feasible for this study	Bespoke NWEH costing: *£11027 data handling* *£1575 data check £1326 project manager* Apollo Medical costing: *£7200 data query development* CK Aspire costing: *£6800 GP recruitment*
The Driver and Vehicle Licensing Agency (DVLA)	*October 2014*: multiple attempts at contact to discuss the feasibility of the study, including telephone calls and email correspondence. No response received *February 2015*: following discussion with a member of a DVLA expert committee, the DVLA medical advisor was contacted. The study was discussed with the DVLA data-sharing team and the response indicated that the DVLA would not have the capacity to assist with the study and the data-security requirements are ‘over and above the NHS or university’	N/A
The Department for Work and Pensions (DWP) HM Revenue and Customs (HMRC)	*November 2014*: first contact regarding feasibility of accessing DWP and HMRC data for this study. Request transferred to the DWP External Data Sharing and Advice Centre *December 2014*: External Data Sharing Advice Centre responded. Data access directly with the DWP or HMRC would not be possible and my request should be redirected to ADRN	N/A
The Administrative Data Research Network (ADRN)	*December 2014*: first contact regarding feasibility for this study. No response received *Feb 2015*: further contact regarding feasibility of the study. General information provided via email *March 2015*: informative teleconference to discuss the study. ADRN confirmed that the study is eligible for their service and they can request access to the DWP/HMRC linked to clinical datasets, such as HES, provided by HSCIC. They agreed to contact the relevant data sources to determine the feasibility *April 2015*: further teleconference, no significant progress *May 2015*: further teleconference, HMRC have declined participation, the DWP remains pending. I am informed that if the DWP does not permit access to its data I cannot apply through ADRN solely for clinical datasets and independent applications must be submitted to the relevant organisations such as HSCIC *July 2015*: informed that the DWP have not been forthcoming but negotiations are on-going and they are unlikely to have a confirmed response until September. No further feedback received	N/A

#### Clinical routine data sources

Routinely recorded secondary-care data can be requested on an individual-level, identifiable basis for patients in England and Wales through HSCIC and NWIS, accessed through SAIL and in our experience this process is feasible as part of a RCT, yet there are notable limitations. In England, HSCIC has set a target time to data access of sixty working days following submission for a complex application, involving bespoke data linkage from multiple datasets. From the date of submission of the Data Access Request Service online application, we have been granted access to the data within this timeframe. However, this positive experience following submission of the application is countered by limitations in the pre-application process. Acknowledging the significant update to online application and approval procedures that occurred during this period, there remains a considerable period of time required in the development of the application. The nature of the request for identifiable data necessitated participant consent as the valid legal basis. HSCIC require ethical and governance approval to be in place prior to DARS review and to prevent future amendments and delays, it was rational to ensure the consent materials had been reviewed by the HSCIC’s Information Governance Team, prior to submitting the documents for ethical and governance approval. HSCIC provide written guidance regarding the consent materials and advise that documents should be reviewed. However, in our experience there is no formalised process for providing this review. Following significant correspondence the consent materials were reviewed by the Data Access and Information Sharing Team. However, this feedback was provided following a formal submission and review by the Data Access Request Service. Formalising the process for the review of consent materials would likely improve the time and resource efficiency for both HSCIC and the researcher.

For participants in Wales, we have requested secondary-care data and, for a proportion of participants, primary-care data through SAIL databank. SAIL provided a streamlined pre-application service, including engaging in multiple discussions and completion of a scoping document outlining the study methods and costs involved. Consent materials were also promptly reviewed by a member of the Information Governance Team.

Common to both sources of secondary-care, routinely recorded data; there are stringent information governance requirements that must be in place prior to application. These include information security measures and assessments, specific inclusion regarding the ‘processing of health care data for the subjects of research’ in the institutional Data Protection Act registration and, in the case of HSCIC, an institutional Data Sharing Framework Contract. Adequate guidance is provided by the data sources and, if not addressed by the researcher, may cause delay. Furthermore, there is a time lag of approximately 3–6 months before data become available within each data source. This delay potentially limits the utility of such sources in prospective clinical research, such as drug trials, where prompt reporting is clinically important and a regulatory requirement.

Routinely recorded primary-care data for specific participants in England are less accessible. The majority of providers of primary-care data, such as ResearchOne and QResearch, provide data on a de-identified basis with no facility to re-identify individuals. Therefore, where specific participants need to be identified, as for RCTs such as SANAD II, these sources are not applicable. Following our correspondence, CPRD confirmed it may be possible to retrieve identifiable individual-level data linked to HSCIC data in the future, but the required approvals were not in place and the timescale to resolution was unclear. Furthermore, such primary-care sources provide data for only a proportion of the population and can be expensive.

North West eHealth employs an alternative methodology whereby primary-care data are extracted directly from the GP through a third party. This process requires participant and GP consent and installation of the required software but is an effective data-extraction method [14]. NWEH offers a number of primary-care research tools for the wider research community but does not currently routinely provide a bespoke primary-care data-extraction service for research.

#### Non-clinical routine data sources

Aggregate economic and societal statistics, provided by Lower Layer Super Output Area (LSOA), can be accessed through the ONS and are in the public domain. Such data may have additional benefits to the analyses of health and socioeconomic outcomes in RCTs. Individual-level, economic data from sources such as the DWP and HMRC would likely be informative to prospective clinical research as such data are often poorly or incompletely recorded using standard methods [15]. However, relevant to this study, there is no previous evidence of access to DWP or HMRC individual-level or aggregate data for clinical research.

During scoping discussions with DWP and HMRC, we were directed to ADRN but this network has not been successful in negotiating data access.

Finally, the outcomes of selected clinical studies may be measured using DVLA data. However, the DVLA declined the request for access, citing insufficient internal resources to process the request and more stringent data protection requirements than those employed in the NHS or academic institutions, without providing explicit details regarding these requirements.

## Discussion

Routinely recorded data are valid for use in retrospective clinical research [3, 4] and have the potential to be used in prospective research including measuring the outcomes of RCTs [7] and providing additional benefits such as a method to address missing RCT data. Limitations, specifically with respect to accuracy and access have been recognised for some time. Academic, political [9] and health service [10] interest in UK sources of routinely recorded data has resulted in expansion and improvements, notably in the access to linked datasets. However, our experience with accessing individual-level data for specific participants providing written consent, to inform the outcomes of a RCT, highlights persisting limitations.

Clinical routine data sources are numerous and there is comprehensive national coverage of secondary-care data. In our experience, accessing individual-level data is feasible. However, inefficiencies in the application processes persist, particularly during the informal ‘pre-application’ phase. The notable limitation encountered was obtaining feedback on the Patient Information Sheet and Consent Form prior to ethical and governance review. Formalising an explicit review process for consent materials would improve the efficiency for both the data holders and the research team.

Access to routinely recorded, individual-level, primary-care data has not been feasible. Each primary-care data source has limited geographical coverage, often based on GP IT systems, which usually process de-identified data and may incur significant expense. The inception of the HSCIC *General Practice Extraction Service*, which records primary-care data nationally for England, represents the most optimistic national source; however, access is currently restricted to Department of Health initiatives such as research involving screening procedures [16].

The access to non-clinical data sources for clinical research has not been possible. ADRN has been established to act on behalf of the researcher in negotiating access to de-identified, linked, routinely recorded data from a number of organisations and the study proposal was promptly directed to ADRN. However, the decision whether to release data remains with the data holder. Ideologically, the next step would be the storage of de-identified linked data from participating organisations in a single repository, similar to those established for RCT data [17]. This would create a single point of access and remove the burden for each organisation to consider each study individually. This would, however, require significant information governance and security barriers to be cleared and, in light of recent developments within the research climate, individual consent. Including patients as stakeholders in the development of such data sources is essential [18].

Although there are examples of pragmatic RCTs being coordinated through routine data sources [8], there are likely to be limitations when accessing routinely recorded data to measure the outcomes of RCTs. Quality assurance is unclear and the level of agreement of routinely recorded data with data recorded through standard RCT methods remains uncertain, particularly when measuring clinical outcomes. The time delay before routinely recorded data become available may have implications for RCTs where prompt reporting is both clinically important and a regulatory requirement. Furthermore the pre-application and application process may introduce further delays. This will have implications for RCTs relying on routinely recorded data. The cost-efficiency of accessing routinely recorded data, compared to standard methods, is unclear. Further research is required to assess the agreement, additional benefits and cost-efficiency of routinely recorded data compared to data collected through standard RCT methods; it may be in the additional benefits, such as addressing missing RCT data, where routinely recorded data is most useful.

## Conclusions

The failure of access to routinely recorded data for a purpose, such as this study with clear secondary benefit to clinical research methodology, seems inappropriate when the ‘public purse’ funds the research, the researcher and the public body holding the data. Perhaps a significant cause or contributor to the current limitations is the Care.Data initiative in 2014. The proposal to extract primary-care records from all patients was opposed publicly by a number of groups and, for example, resulted in an internal inquiry within HSCIC. Data applications were suspended during this period and our current experience may be explained by the concurrent revision of the HSCIC application and approval procedures. However, in the medium term, of more concern is the harm in public perception that resulted. Currently, more than 1.2 million individuals in the UK have submitted a ‘Type 2 objection’, meaning that their data will not be shared for purposes other than direct care [19]. Although the application procedures may improve, and in time we may be able to access data more efficiently, the loss of 2.2% of the population’s data will have implications for the routinely recorded data that will then be made available for research. Involving patients as important stakeholders and re-gaining their trust will be an essential factor in realising the individual and population health care benefits of routinely recorded data [20].

### Recommendations

We propose recommendations to improve access and implementation of routinely recorded data during a RCT, summarised in Table 5.

**Table 5:** Recommendations to improve access to routinely recorded data for research

*General*
Routinely recorded data are being used to measure randomised controlled trial (RCT) outcomes with the agreement, additional benefits and cost-efficiency of such data compared to data recorded through standard RCT methods being unknown *Further research should be performed to assess the agreement, additional benefits and cost-efficiency of accessing routinely recorded data to measure RCT outcomes compared to data collected through standard RCT methods*
The costs required for data access from routine data sources vary widely, although all reportedly operate on a cost recovery, not-for-profit basis *Costs should be standardised and rationalised between routine data sources*
The time lag before data are available in routine data sources represents a significant limitation to the access of routinely recorded data for prospective research, including RCTs *The infrastructure and procedures should be developed to reduce the time lag seen in routinely recorded data sources*
The requirement for linkage between sources of routinely recorded data has been observed and improvements are on-going; for example, with the establishment of the Administrative Data Research Network (ADRN) *A standardised set of identifying variables could be recorded by all (clinical and non-clinical) data sources to improve the accuracy of data linkage, similar to a Core Outcome Set for clinical trials* [44]
The public mistrust in the sharing and linking of routinely recorded data will hamper future efforts to develop routinely recorded databases, despite the likely benefits to individual patients and the population *Further research and public engagement should be undertaken to define the issues of most importance to the public and develop strategies to address these*
*Clinical routine data sources*
There are numerous requirements prior to application, and criteria to fulfil on submission, of an application, yet the guidance and support during development of an application remains limited *Formalise and improve access to guidance and review of study materials during the ‘pre-application stage’*
There is national coverage of routinely recorded secondary-care data, yet primary-care coverage remains patchy, based on geographical area or GP IT system *Develop the primary-care data sources to provide national coverage, either through collaboration of existing sources and data linkage or development of national data sources, such as the General Practice Extraction Service*
*Non-clinical routine data sources*
Access to non-clinical data sources to inform clinical research was not possible during this study, despite the significant potential to inform Health Technology Assessment and the increasing importance of such assessments in a health care system where resources are increasingly limited *To assist with Health Technology Assessment, and particularly the analysis of health economic outcomes, urgent research is required to consider facilitating access to individual-level, identifiable data from non-clinical sources. This would include*: 1. *Research regarding the public perception and acceptability of using their personal economic data for clinical research* 2. *Internal review within non-clinical sources, such as the DWP and HMRC, to assess the feasibility and limitations of permitting access to data for clinical research* 3. *Formalisation of the approval processes through the independent party, the ADRN for access to non-clinical administrative data – currently, following internal approval the ADRN then negotiates access to administrative data on a project-by-project basis*

## Abbreviations

ADRN: Administrative Data Research Network
CPRD: Clinical Practice Research Datalink
DVLA: Driver and Vehicle Licensing Agency
DWP: Department for Work and Pensions
HMRC: HM Revenue and Customs
HSCIC: The Health and Social Care Information Centre
ISD: Information Services Division
NHS: National Health Service
NWEH: North West eHealth
NWIS: NHS Wales Information Centre
ONS: The Office for National Statistics
RCT: Randomised controlled trial
SAIL: The Secure Anonymised Information Linkage Databank
SANAD II: The Standard and New Antiepileptic Drugs II trial
THIN: The Health Improvement Network
